# Effect of environmental stress factors on the expression of virulence genes and pathogenicity of lethal *Bacillus cereus* of bovine origin

**DOI:** 10.3389/fmicb.2025.1519202

**Published:** 2025-02-13

**Authors:** Qinglei Meng, Lili Song, Shanshan Chi, Haifeng Wang, Jie Li, Yunjiao Chen, Zhilin Liu, Xin Zhang, Zelin Jia, Jiayu Cui, Xueli Wang

**Affiliations:** ^1^College of Animal Science and Technology, Inner Mongolia Minzu University, Tongliao, China; ^2^Department of Grassland Ecology and Animal Husbandry and Veterinary, Xilingol Vocational College, Xilinhot, China; ^3^Tongliao Institute of Agriculture and Animal Husbandry Sciences, Tongliao, China; ^4^Hure Banner Animal Disease Prevention and Control Center, Tongliao, China

**Keywords:** environmental stressors, lethal *B. cereus*, fluorescence quantitative PCR, expression of virulence genes, pathological sections

## Abstract

**Objective:**

*Bacillus cereus* (*B. cereus*) can be used as a probiotic or produce a variety of toxins that are pathogenic to humans and animals. Environmental stressors can affect the growth process of *B. cereus* and the expression of its virulence genes. Due to the limitations of methods such as pharmacological disinfection methods (there are limits to the use of antibiotics) and chemical disinfection methods (chemical methods may produce residues), attempts can be made to remove and reduce *B. cereus* infections through environmental stress factors.

**Methods:**

In this study, the expression of four virulence genes (*nheA*, *hblD*, *cytK*, and *entFM*) of bovine-origin lethal *B. cereus* was investigated by qPCR under the effect of different environmental stressors. The extent of pathological damage to various organs of mice by *B. cereus* was observed by pathological sections.

**Results:**

The results showed that high temperature could inhibit the expression of *B. cereus* virulence genes. Expression of *B. cereus* virulence genes was affected under the influence of pH. Different salt concentrations could make the *B. cereus* virulence genes show low expression. Under a single environmental stressors, *nheA*, *hblD*, *cytK*, and *entFM* had the lowest expression at 40°C, pH 8.0, and were lowly expressed at all salt concentrations except the control group. The action of multiple environmental stressors affect the expression of virulence genes. Under multiple environmental stressors, *nheA*, *hblD* and *cytK* were least expressed at a temperature of 40°C, pH 6.0, and salt concentration of 3.0%, and *entFM* was least expressed at a temperature of 20°C, pH 8.0, and salt concentration of 1.5%. Animal pathogenicity tests have shown that environmental stressors affect the virulence of *B. cereus*.

**Conclusion:**

The level of virulence gene expression in *B. cereus* can be reduced by environmental stress factors, thus further reducing the risk of *B. cereus* to human health. This study provides some reference for the prevention and control of *B. cereus* disease.

## 1 Introduction

*B. cereus* is one of the more common conditionally pathogenic bacteria in the family *Bacillaceae*. Some *B. cereus* can be used as plant growth promoters and microbial additives for animal feeds (Elsayed et al., 2022; Gong et al., 2018). *B. cereus* produces vomiting enterotoxins and diarrhea enterotoxins that cause disease in humans and animals (Jia et al., 2022). Pathogenic *B. cereus* produces toxins that in turn cause disease in humans and animals, and in a few cases can be life-threatening. *B. cereus* is an increasing hazard to human health, and controlling the means of transmission of *B. cereus* can reduce *B. cereus* infections. The removal and reduction of *B. cereus* from the environment is important to ensure the safety of the food handling environment. *B. cereus* produces spores that are thermally stable, a property that allows *B. cereus* to survive in harsh environments (Bai et al., 2018). *B. cereus* is subject to acidity stress (including the combined effects of inorganic and organic acid stress) in environments with low pH. It has been shown that *B. cereus* has a lag period of 1, 2, and 5 h for experimental strains at pH 7.0, 5.3, and 4.9, respectively (Biesta-Peters et al., 2011). Other studies have shown that *B. cereus* can reproduce normally in higher salt concentrations (6.5–7.5%) (Jeon et al., 2016). In response to salt stress in *B. cereus*, the increase in Na^+^ and H^+^ components of the osmoprotectant and the activation of the oxidative stress response may create cross-protection against other stresses (Belaouni et al., 2022).

In this paper, four virulence genes (*nheA*, *hblD*, *cytK*, and *entFM*) of *B. cereus* were selected as targeted genes (it has been shown in the literature that these four virulence genes have high detection rates and high toxicity) (Chang et al., 2021; Berthold-Pluta et al., 2019; Rossi et al., 2018). The NHE complex contains three different proteins, NheA, NheB, and NheC, which are encoded by the *nheA* gene, the *nheB* gene and the *nheC* gene, respectively. Of these, NheA is considered to be the major diarrheal enterotoxin of *B. cereus*. The HblD is hemolytic and cytotoxic to intestinal and other cells. CytK is associated with foodborne diarrhea caused by *B. cereus* strains, it is cytotoxic and hemolytic, and the toxin may cause necrotizing enterocolitis in animals. EntFM is known as a cell wall peptidase and is involved in adhesion, motility, biofilm formation and cytotoxicity of *B. cereus*. In the current study, the relative expression of *B. cereus* virulence genes after 14 h of incubation under different stress conditions were determined by qPCR. A mouse model was used to evaluate the pathological damage caused by *B. cereus* to various organs of mice after 14 h of incubation under different stress conditions. The aim of this study was to identify environmental conditions from experimental data that are relatively unfavorable for the growth and expression of virulence genes in *B. cereus*. In this study, we controlled the expression levels of bacterial virulence genes through environmental stressors to reduce *B. cereus* infections in humans and animals. Prevention and control of *B. cereus* disease by controlling environmental stress factors is of clinical significance. This method can be used to prevent and control *B. cereus* disease by reducing the expression of *B. cereus* virulence genes. Common sterilization methods have limitations (for example, both pharmaceutical and chemical disinfection methods can produce residues; chemical disinfection methods have a higher odor, etc.). The method used in this study does not produce residues and is relatively safe to operate. This study is expected to provide some ideas for the prevention and control of *B. cereus* disease in the future.

## 2 Materials and methods

### 2.1 Test materials

Luria-Bertani (LB) agar medium (tryptone 10 g/L, yeast extract 5 g/L, sodium chloride 10 g/L, agar powder 15 g/L), LB broth medium (tryptone 10 g/L, yeast extract 5 g/L, sodium chloride 10 g/L). Sodium chloride, nutrient agar, glucose, and agar powder were purchased from Beijing Chemical Factory; yeast extract, tryptone were purchased from Zhengzhou Dening Biotechnology Company; multifunctional constant temperature shaker, constant temperature incubator were purchased from Henan Wolin Instrument Company; RNAprep pure Bacteria Kit, Bacterial genomic DNA extraction kit, TB Green^®^ Premix Ex Taq™ II (Tli RNaseH Plus), DL2000 DNA marker, PCR mastermix, nucleic acid dye, agarose, 50 × Tris Acetate-EDTA buffer were purchased from TaKaRa Company (Bai et al., 2018; Li, 2017). Automatic gel imaging analysis system ZF-258 was purchased from TwisDxTM Company; StepOnePlus™ Real-Time Fluorescence PCR System and Evolution™ Pro UV–Vis spectrophotometer were purchased from Thermo Fisher Company. *B. cereus* strain was kindly provided by Laboratory of Veterinary Pathology, College of Animal Science and Technology, Inner Mongolia Minzu University, and the strain (we named the strain lycx) was isolated from the spleens of the sudden death cattle. A total of 200 Kunming mice (6–8 weeks old, 25–30 g weight) were purchased from Liaoning Changsheng Biotechnology Company. McFarland turbidimeter (Bacterial turbidimeter), McFarland turbidimeter tube were purchased from Shanghai Kunquan Biotechnology Company. The bacterial genomic DNA of experimental strains were uploaded in the sequence of the experimental strain to GenBank (Serial numbers are CP129005.1 and CP129006.1). Isolates were deposited at China General Microbiological Culture Collection Center (CGMCC No.17626).

### 2.2 Methods

#### 2.2.1 Plotting the growth curve of *B. cereus* strain

*B. cereus* was incubated with LB broth medium for 14 h with shaking (37°C, 160 rpm), and the OD value of the bacterial culture was measured every 2 h and the growth curve of the bacteria was plotted (Use of GraphPad Prism version 5.01). Cultures of *B. cereus* were carried out in three replicates.

#### 2.2.2 Detection of virulence genes in *B. cereus*

DNA of the strain was extracted using a Bacterial genomic DNA extraction kit. Primers were synthesized with reference to the relevant literature (as shown in Table 1) and detected using agarose gel electrophoresis after PCR amplification (Hansen and Hendriksen, 2001; Ngamwongsatit et al., 2008; Agata et al., 1995; Asano et al., 1997; Kim et al., 2013; Wang and Liu, 2018).

**Table 1:** *B. cereus* virulence genes primer sequences and qPCR primer sequences.

(A) *B. cereus* virulence gene primer sequences.				

Virulence gene	Primer sequences (5′–3′)	Product length (bp)	Annealing temperature (°C)	References
*nheA*	F: TACGCTAAGGAGGGGCA	500	57	Hansen and Hendriksen (2001)
	R: GTTTTATTGCTTCATCGGCT			
*nheB*	F: CAAGCTCCAGTTCATGCGG	935	56	Ngamwongsatit et al. (2008)
	R: GATCCCATTGTGTACCATTG			
*nheC*	F: ACATCCTTTTGCAGCAGAAC	618	56	Ngamwongsatit et al. (2008)
	R: CCACCAGCAATGACCATATC			
*hblA*	F: GCAAAATCTATGAATGCCTA	884	54	Ngamwongsatit et al. (2008)
	R: GCATCTGTTCGTAATGTTTT			
*hblC*	F: CCTATCAATACTCTCGCAA	695	54	Ngamwongsatit et al. (2008)
	R: TTTCCTTTGTTATACGCTGC			
*hblD*	F: AATCAAGAGCTCTCACGAAT	430	54	Hansen and Hendriksen (2001)
	R: CACCAATTGACCATGCTAAT			
*bceT*	F: TTACATTACCAGGACGTGCTT	428	56	Agata et al. (1995)
	R: TGTTTGTGATTGTAATTCAGG			
*cytK*	F: CGACGTCACAAGTTGTAACA	565	54	Ngamwongsatit et al. (2008)
	R: CGTGTGTAAATACCCCAGTT			
*entFM*	F: ATGAAAAAAGTAATTTGCAGG	1269	56	Asano et al. (1997)
	R: TTAGTATGCTTTTGTGTAACC			
*ces*	F: GCATTTCGTGAAGCAGAGGT	699	54	Kim et al. (2013)
	R: CCCTTTATCCCCTTCGATGT			
*groEL*	F: AGCTATGATTCGTGAAGGT	236	54	Kim et al. (2013)
	R: AAGTAATAACGCCGTCGT			
*plcR*	F: AAAAAGGAAGAATATCATC	424	52	Wang and Liu (2018)
	R: ATGCATCTTCAATCTCTG			

(B) qPCR primer sequences.				
Virulence gene	qPCR primer sequences (5′–3′)	Product length (bp)	Annealing temperature (°C)	References
*gat B_Yqey*	F: AGCTGGTCGTGAAGACCTTG	175	60	Reiter et al. (2011)
	R: CGGCATAACAGCAGTCATCA			
*nheA*	F: CTAAGGAGGGGCAAACAGAAG	117	60	Li (2017)
	R: CGTATGCTTGAATAAGCGGTG			
*hblD*	F: CCCCGAAGGACTAAAGAAAGC	128	60	Li (2017)
	R: CCCCCTCTGAGCCTAAATTGAT			
*entFM*	F: ATTGCAGGTTTAGCAGCAGCTT	85	60	Li (2017)
	R: GCGCTTCATTTGAAACTTGTGC			
*cytK*	F: GCGCTGATAAACAGATTGCCGT	105	60	Li (2017)
	R: TAGCGCCAGGGATTGGGTAGTT			

#### 2.2.3 Experimental design for culturing *B. cereus* strain and gene expression analysis under varying conditions

*B. cereus* was cultured (37°C, 14 h) using LB broth medium. Temperature group medium: LB broth medium was incubated at 20, 30, and 40°C, and the experimental groups were named as “group 20,” “group 30,” and “group 40.” pH group medium: The pH of LB broth medium was adjusted to 6.0, 7.0, and 8.0, and the experimental groups were named as “group 6,” “group 7,” and “group 8.” Salt concentration group medium: The salt concentration of LB broth medium was adjusted to 0, 1.5, and 3.0%, and the experimental groups were named as “group 0,” “group 1.5,” and “group 3.” Total RNA was extracted from the strains using an RNA extraction kit, and the RNA concentration was determined and then the RNA was reverse transcribed to cDNA. Synthetic primers were designed according to the relevant literature (as shown in part b of Table 1; Xu, 2023; Jing et al., 2018). The cDNA was used as a template, and PCR amplification of the *gatB_Yqey* gene was verified by agarose gel electrophoresis (Reiter et al., 2011). The expression of four virulence genes, *nheA*, *hblD*, *entFM*, and *cytK*, was determined using *gat B_Yqey* as an internal reference gene. Reactions were performed using qPCR kits (3 replicates of qPCR reactions were performed for each test group) and experimental data were saved for subsequent analysis (Use of GraphPad Prism version 5.01).

#### 2.2.4 Effects of multifactorial environmental stressors on the expression of virulence genes in *B. cereus*

Using an orthogonal test, a 3-factor (temperature, pH, salt concentration), 3-level manipulation was designed (Wang and Liu, 2018). The factors and levels are shown in part “a” of Table 2, and the operational protocol for the orthogonal test is shown in part “b” of Table 2. Experimental data were analyzed using GraphPad Prism version 5.01. It has been shown that the optimum growth temperature of *B. cereus* is 28–35°C (Reiter et al., 2011). We chose three temperatures, 20, 30, and 40°C, to conduct the experiment in this study. *B. cereus* grows more slowly when temperatures are too high or too low, and it is less harmful to humans and animals. Have a neutral pH, so gradients of 6.0, 7.0, and 8.0 were chosen for the test. Bacteria grown under mild salt stress conditions showed a slight decrease in growth rate compared to normal growth conditions. Severe salt stress may lead to a lag period in bacterial growth followed by a resumption of growth, so the salt concentration used should not be too high (Li, 2017).

**Table 2:** Factor levels and experimental program for orthogonal testing of environmental stress factors.

(A) Orthogonal test factor levels for environmental stressors.			
Level	Factor		
	A: Temperature (°C)	B: pH	C: Salt concentration (%)
1	20	6.0	0
2	30	7.0	1.5
3	40	8.0	3.0

(B) Orthogonal test protocol for environmental stressors.			
Serial number	A: Temperature (°C)	B: pH	C: Salt concentration (%)
11	40	8.0	0
12	20	7.0	3
13	40	6.0	3
14	20	8.0	1.5
15	30	8.0	3
16	40	7.0	1.5
17	30	7.0	0
18	30	6.0	1.5
19	20	6.0	0

Total bacterial RNA was extracted and reverse transcribed into cDNA using *B. cereus* cultured under the above conditions. The quality of cDNA was assessed through endpoint PCR targeting the housekeeping gene *gatB_Yqey*, followed by analysis using agarose gel electrophoresis. The validated cDNA was then utilized for qPCR reactions.

#### 2.2.5 Effects of environmental stressors on the pathogenicity of *B. cereus*

Effect of unifactorial environmental stressors on the pathogenicity of *B. cereus*. Mice were given intraperitoneal injections of *B. cereus* bacterial culture (Each mouse was injected intraperitoneally with 0.3 mL of bacterial culture at a concentration of 10^9^ CFU/mL) in 9 groups (groups 0, 1.5, 3, 6, 7, 8, 20, 30, 40). There were 10 mice in each group. Twenty more mice were also selected as a control group (10 injected with LB broth medium; 10 injections of bacterial culture cultured using the conditions of 1.2.1, with a salt concentration of 1%, a pH of 7 and an incubation temperature of 37°C). The salt concentrations were 0, 1.5, and 3.0% in groups 0, 1.5, and 3, respectively; in the remaining groups the salt concentration was 1%. Groups 6, 7, 8 corresponded to the following culture media pH: 6, 7, and 8, respectively; the rest of the groups had pH 7.2. Groups 20, 30, and 40 were incubated at 20, 30, and 40°C, respectively; the remaining groups were incubated at 37°C. The time of death was recorded for each group of mice. Mice were dissected immediately after death. Mice that did not die after 3 d (d: day) were anesthetized with 0.3% pentobarbital (5 mg/100 g, intraperitoneal injection), whereas the mice were then executed using the cervical dislocation method and immediately dissected after execution (National Research Council (US) Committee for the Update of the Guide for the Care and Use of Laboratory Animals, 2011). The organs of dissected mice were immersed and fixed for 3 days in 10% formaldehyde solution. Pathologic tissue sections were made using mouse organs (Hematoxylin–Eosin staining).

Effect of multifactorial environmental stressors on the pathogenicity of *B. cereus*. Mice were given intraperitoneal injections of *B. cereus* bacterial culture (groups 11, 12, 13, 14, 15, 16, 17, 18, 19), 10 in each group. The salt concentrations of groups 11, 12, 13, 14, 15, 16, 17, 18, and 19 were 0, 3.0, 3.0, 1.5, 3.0, 1.5%, 0, 1.5%, and 0, respectively. The pH of each group was 8.0, 7.0, 6.0, 8.0, 8.0, 7.0, 7.0, 6.0, and 6.0, respectively. The incubation temperatures were 40, 20, 40, 20, 30, 40, 30, 30, and 20°C, respectively. Pathologic tissue sections were made using mouse organs (Hematoxylin–Eosin staining). Mice were executed in the same way as in the previous paragraph. The organs of the mice were handled in the same way as in the previous paragraph.

## 3 Results

### 3.1 Results of plotting *B. cereus* growth curve

The genome sequence number of the test strain was CP129005.1 and the genome size was 5,484,835 bp (GC content 35.39%). The plasmid sequence of the test strain was CP129006.1 with a plasmid size of 609,501 bp (GC content of 32.16%).

**Figure 1:**
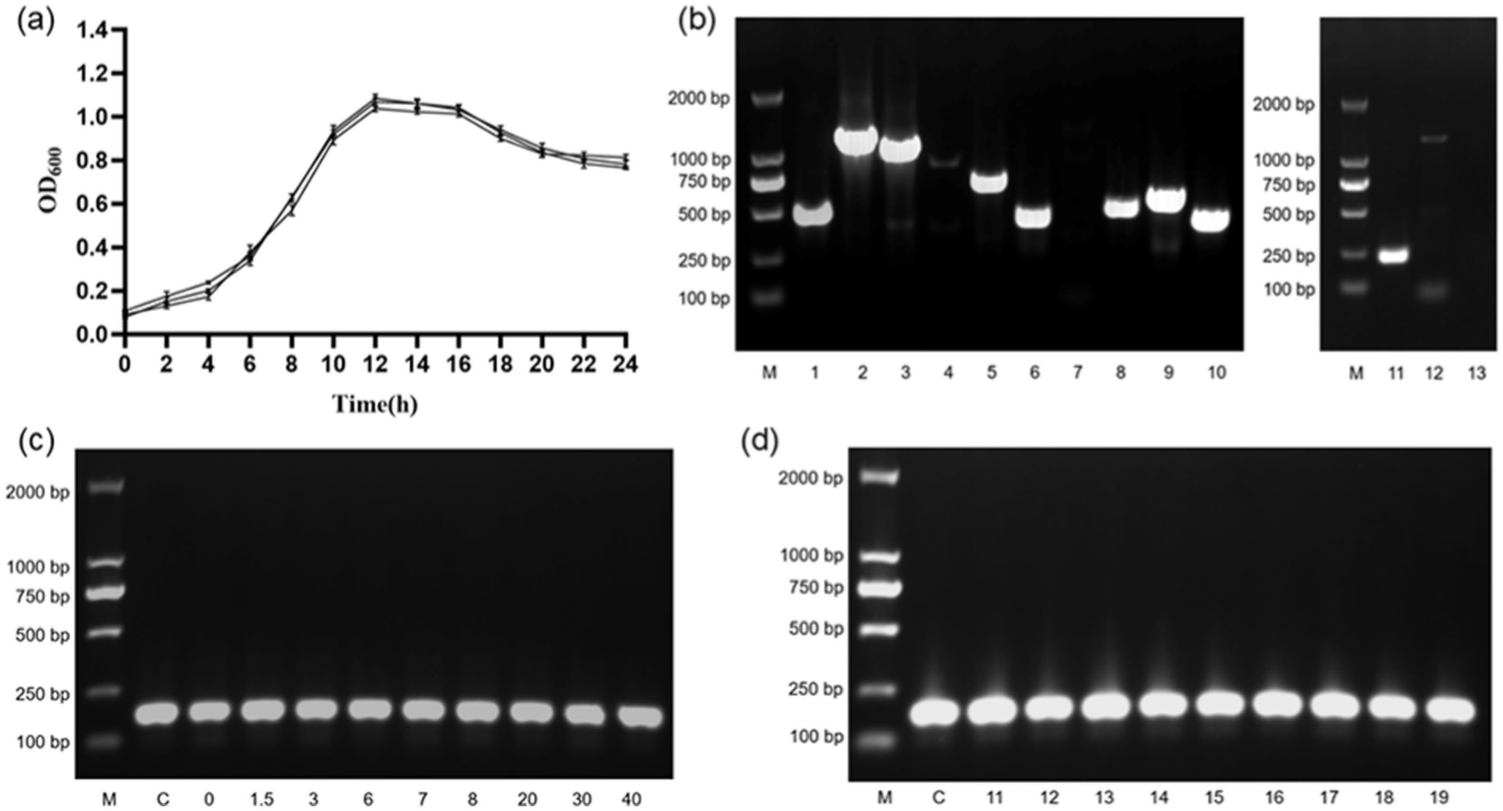
*B. cereus* growth curves and genetic validation results. **(A)** 1–24 h growth curve of *B. cereus*. **(B)** Results of the *B. cereus* virulence gene assay. M, DL2000 DNAMarker; 1, *nheA* gene; 2, *nheB* gene; 3, *nheC* gene; 4, *hblA* gene; 5, *hblC* gene; 6, *hblD* gene; 7, *ces* gene; 8, *plcR* gene; 9, *cytK* gene; 10, *bceT* gene; 11, *groEL* gene; 12, *entFM* gene; 13, negative control. **(C)** RNA validation of single factor stressors. M, DL2000 DNAMarker; C, Control group; 0, group 0; 1.5, group 1.5; 3, group 3; 6, group 6; 7, group 7; 8, group 8; 20, group 20; 30, group 30; 40, group 40. **(D)** RNA validation of multifactorial stressors. M, DL2000 DNAMarker; C, Control group; 11, group 11; 12, group 12; 13, group 13; 14, group 14; 15, group 15; 16, group 16; 17, group 17; 18, group 18; 19, group 19.

Growth curves of *B. cereus* over 24 h were plotted (as shown in Figure 1A). Incubation time was taken as the horizontal coordinate and OD_600_ as the vertical coordinate. The results showed that *B. cereus* had slow cell division in 0–2 h (lag phase), rapid cell division in 2–12 h (logarithmic phase), cell division reached the peak of reproduction at 12 h (stationary phase), followed by a slowdown of cell division (decline phase), and gradual cell death after stabilization in 20–24 h.

### 3.2 Results of the *B. cereus* virulence gene assay

The results (as shown in Figure 1B) showed that the house-keeping gene *groEL*, the hemolytic enterotoxin genes *hblA*, *hblC* and *hblD*, the nonhemolytic enterotoxin genes *nheA*, *nheB* and *nheC*, the enterotoxin genes *bceT* and *entFM*, the cytotoxin gene *cytK*, and the pleiotropic regulator *plcR* were presence in our *B. cereus* strain. The vomitoxin gene *ces* was not presence in our *B. cereus* strain.

### 3.3 qPCR results under the influence of single factor environmental stressors

The cDNA obtained from the reverse transcription reaction was used as a template for PCR amplification of the target gene (*gat B_Yqey*). The target bands (approximately 175 bp) were visible in all groups (as shown in Figures 1C,D). This indicates that *gat B_Yqey* gene expression is stable and suitable for use as an internal reference.

#### 3.3.1 Effect of temperature factors on the expression of virulence genes in *B. cereus*

**Figure 2:**
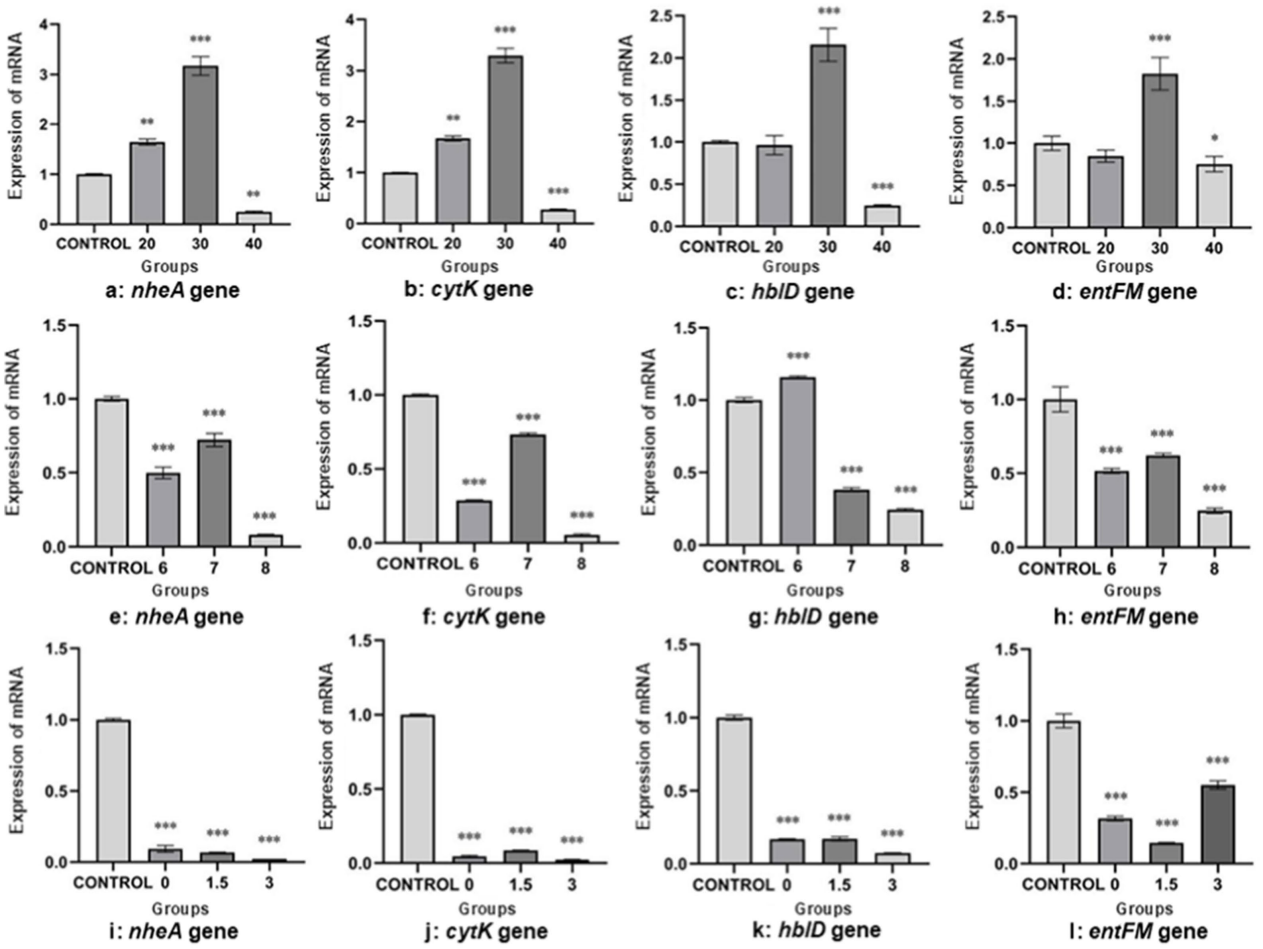
Relative mRNA expression of virulence genes under unifactorial environmental stresses **(A–D)**. The expression of virulence genes (*nheA*, *cytK*, *hblD* and *entFM*) of *B. cereus* in different environments (20, 30, and 40°C), respectively **(A–D)**. The expression of virulence genes (*nheA*, *cytK*, *hblD* and *entFM*) of *B. cereus* at different pH (6.0, 7.0, and 8.0), respectively **(E–H)**. The expression of virulence genes (*nheA*, *cytK*, *hblD*, and *entFM*) of *B. cereus* at different salt concentrations (0, 1.5, and 3.0%), respectively **(I–L)**. The data obtained above are mean ± standard deviation, where *P < 0.05; **p < 0.01; ***p < 0.001 indicates statistically significant differences.

The results of the analysis of the test data are shown in Figures 2A–D. The expression of both *nheA* gene and *cytK* gene were higher than that of the control group when incubated at 20°C, and the difference were significant (*p* < 0.01) compared with that of the control group (37°C). The expression of *hblD* gene and *entFM* gene were lower than that of the control group, and none of them were statistically different from the control group (*p* > 0.05). The expression of the four virulence genes, *nheA*, *cytK*, *hblD* and *entFM*, were higher than that of the control group when incubated at 30°C, and the difference between each group and the control group (37°C) were highly significant (*p* < 0.001). The relative expression of both *cytK* and *hblD* genes were lower than that of the control when cultured at 40°C, and the differences were both highly significant (*p* < 0.001) compared with the control (37°C). The expression of *nheA* gene was lower than that of the control group, and the difference was significant (*p* < 0.01) compared with the control group (37°C). The expression of *entFM* gene was lower than that of the control group and was statistically different (*p* < 0.05) from that of the control group (37°C).

#### 3.3.2 Effect of pH on the expression of virulence genes in *B. cereus*

The results of the analysis of the test data are shown in Figures 2E–H. The *nheA* gene, *cytK* gene and *entFM* gene showed low expression compared to the control group. The expression of *hblD* gene was higher than the control at pH 6.0 and lower than the control at both pH 7.0 and 8.0. The difference between the groups and the control group was highly significant (*p* < 0.001).

#### 3.3.3 Effect of salt concentration factors on the expression of virulence genes in *B. cereus*

The results of the analysis of the test data are shown in Figures 2I–L. The expression of *nheA*, *cytK*, *hblD*, and *entFM* genes were lower than that of the control group (salt concentration of 1%). The difference between the groups and the control group were highly significant (*p* < 0.001).

### 3.4 qPCR results under the influence of multifactorial environmental stressors

#### 3.4.1 Effects of multifactorial environmental stressors on *nheA* gene expression

**Figure 3:**
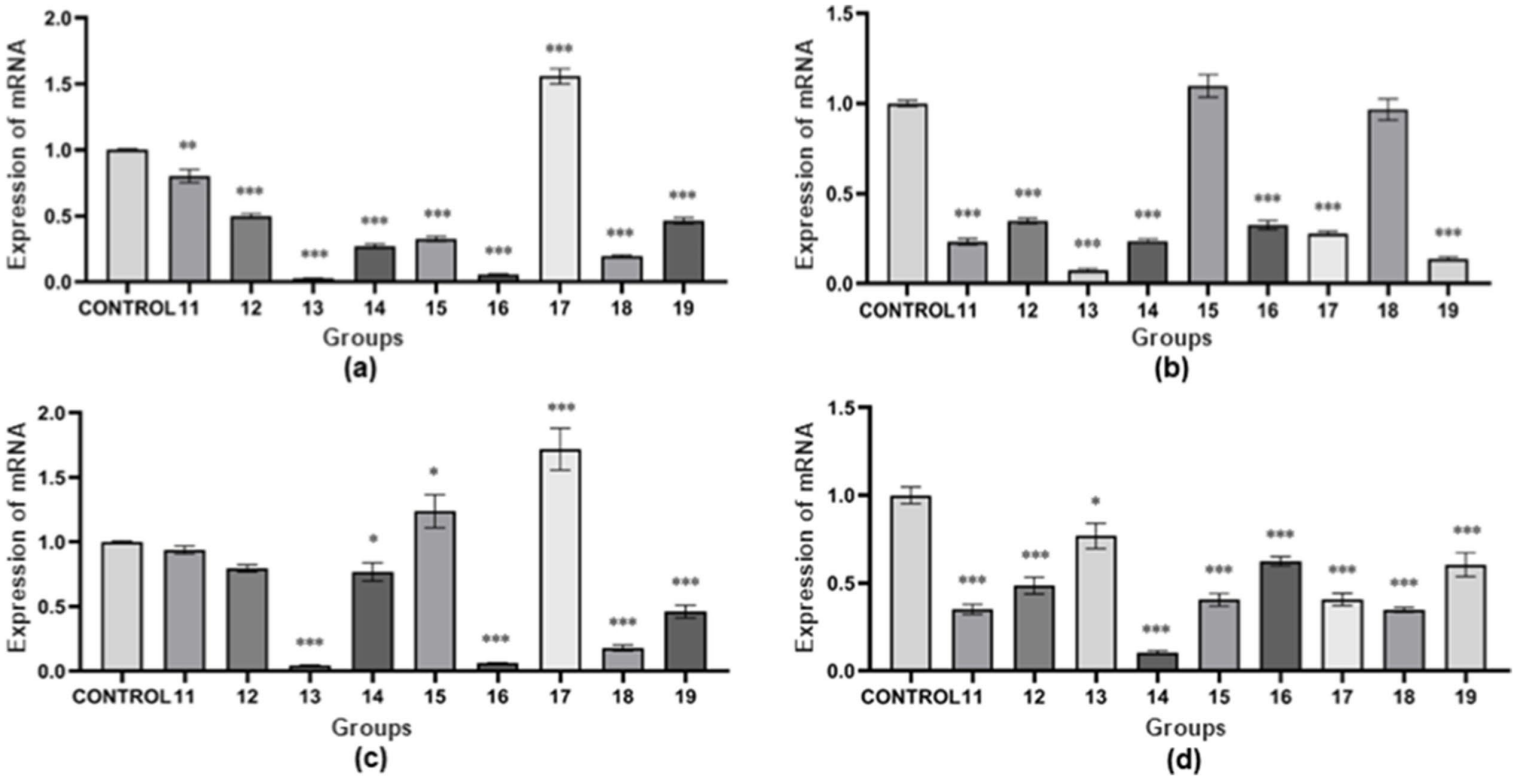
Relative mRNA expression of virulence genes under multifactorial environmental stresses. **(A)** The relative mRNA expression of the *nheA* gene under multifactorial environmental stress. **(B)** The relative mRNA expression of the *hblD* gene under multifactorial environmental stress. **(C)** The relative mRNA expression of the *cytK* gene under multifactorial environmental stress. **(D)** The relative mRNA expression of the *entFM* gene under multifactorial environmental stress. The data obtained above are mean ± standard deviation, where *p < 0.05; **p < 0.01; ***p < 0.001 indicates statistically significant differences.

The results show (as shown in Figure 3A). The expression of *nheA* gene was higher in “group 17” than in the control group, and lower in the other groups than in the control group. In “group 11,” the expression of *nheA* gene was significantly different from that of the control group (*p* < 0.01). In “group 12,” “group 13,” “group 14,” “group 15,” “group 16,” “group 17,” “group 18,” and “group 19,” the expression of *nheA* gene were all highly significantly different from the control group (*p* < 0.001). The results of Type III variance (ANOVA) in orthogonal tests are shown in Table 3A. The effect of the three factors on *nheA* expression was NaCl > pH > temperature (the magnitude of the effect was determined by the *F*-value). There was no significant difference between the three factors (*p* > 0.05).

**Table 3:** ANOVA results of different genes in orthogonal tests.

(A) ANOVA results of *nheA* gene expression.					
Source of variation	Type III variance	df	Mean Square	*F*	*P*
Temperature	0.716	2	0.358	0.441	0.694
pH	1.626	2	0.813	1.003	0.499
NaCl	4.608	2	2.304	2.841	0.260
Error	1.622	2	0.811		

(B) ANOVA results of *hblD* gene expression.					
Source of variation	Type III variance	df	Mean Square	*F*	*P*
Temperature	0.446	2	0.223	0.966	0.509
pH	0.329	2	0.165	0.713	0.584
NaCl	0.007	2	0.003	0.015	0.986
Error	0.462	2	0.231		

(C) ANOVA results of *cytK* gene expression.					
Source of variation	Type III variance	df	Mean Square	*F*	*P*
Temperature	0.571	2	0.286	0.361	0.735
pH	4.395	2	2.197	2.774	0.265
NaCl	4.482	2	2.241	2.829	0.261
Error	1.584	2	0.792		

(D) ANOVA results of *entFM* gene expression.					
Source of variation	Type III variance	df	Mean Square	*F*	*P*
Temperature	0.735	2	0.368	6.916	0.126
pH	0.035	2	0.018	0.333	0.750
NaCl	0.301	2	0.150	2.831	0.261
Error	0.106	2	0.053		

#### 3.4.2 Effects of multifactorial environmental stressors on *hblD* gene expression

The results show (as shown in Figure 3B). The expression of *hblD* gene was higher in “group 15” than in the control group, while the expression in other groups was lower than in the control group. There was no statistically significant difference between “group 15” and “group 18” and the control group (*p* > 0.05). “Group 11,” “group 12,” “group 13,” “group 14,” “group 16,” “group 17,” and “group 19” showed highly significant differences compared to the control group (p < 0.001). The results of Type III variance (ANOVA) in orthogonal tests are shown in Table 3B. The effect of the three factors on *hblD* expression was temperature > pH > NaCl (the magnitude of the effect was determined by the *F*-value). There was no significant difference between the three factors (*p* > 0.05).

#### 3.4.3 Effects of multifactorial environmental stressors on *cytK* gene expression

The results show (as shown in Figure 3C). The expression of *cytK* gene was higher than that of the control group in “group 15” and “group 17,” and lower than that of the control group in the other groups. “Group 13,” “group 16,” “group 17,” “group 18,” and “group 19” showed highly significant differences compared to the control group (*p* < 0.001). “Group 14” and “group 15” were statistically different from the control group (*p* < 0.05). There was no statistically significant difference between “group 11” and “group 12” and the control group (p > 0.05). The results of Type III variance (ANOVA) in orthogonal tests are shown in Table 3C. The effect of the three factors on *cytK* expression was NaCl > pH > temperature (the magnitude of the effect was determined by the *F* value). There was no significant difference between the three factors (*p* > 0.05).

#### 3.4.4 Effects of multifactorial environmental stressors on *entFM* gene expression

The results show (as shown in Figure 3D). The expression of *entFM* gene was lower than that of the control group in all groups. There was a statistically significant difference between “group 13” and the control group (*p* < 0.05). “Group 11,” “group 12,” “group 14,” and “group 15,” “group 16,” “group 17,” “group 18,” and “group 19” showed highly significant differences compared to the control group (*p* < 0.001). The results of Type III variance (ANOVA) in orthogonal tests are shown in Table 3D. The effect of the three factors on *entFM* expression was temperature > NaCl > pH (the magnitude of the effect was determined by the *F*-value). There was no significant difference between the three factors (*p* > 0.05).

### 3.5 Effects of environmental stressors on *B. cereus* pathogenicity

Some mice in groups 20 and 30 in the unifactorial group died within 3–6 h after inoculation with the bacterial culture. Mice in groups 6 and 7 partially died about 6 h after inoculation with the bacterial culture. No mice died in the LB medium control group and 2 mice died in the bacterial culture control group. Three mice died in group 20 and two mice died in group 30. Two mice died in group 6 and three mice died in group 7. One mouse died in groups 40, 8, and 1.5, and no mice died in groups 0 and 3. Some of the mice in groups 11, 15, 17, and 18 of the multifactorial group appeared to die within 3–6 h after inoculation with the bacterial culture, and the numbers of dead mice were 2, 3, 2, and 3, respectively. The number of dead mice in “group 12,” “group 13,” “group 14,” “group 16,” and “group 19” were 1, 0, 0, 1, and 0, respectively.

**Figure 4:**
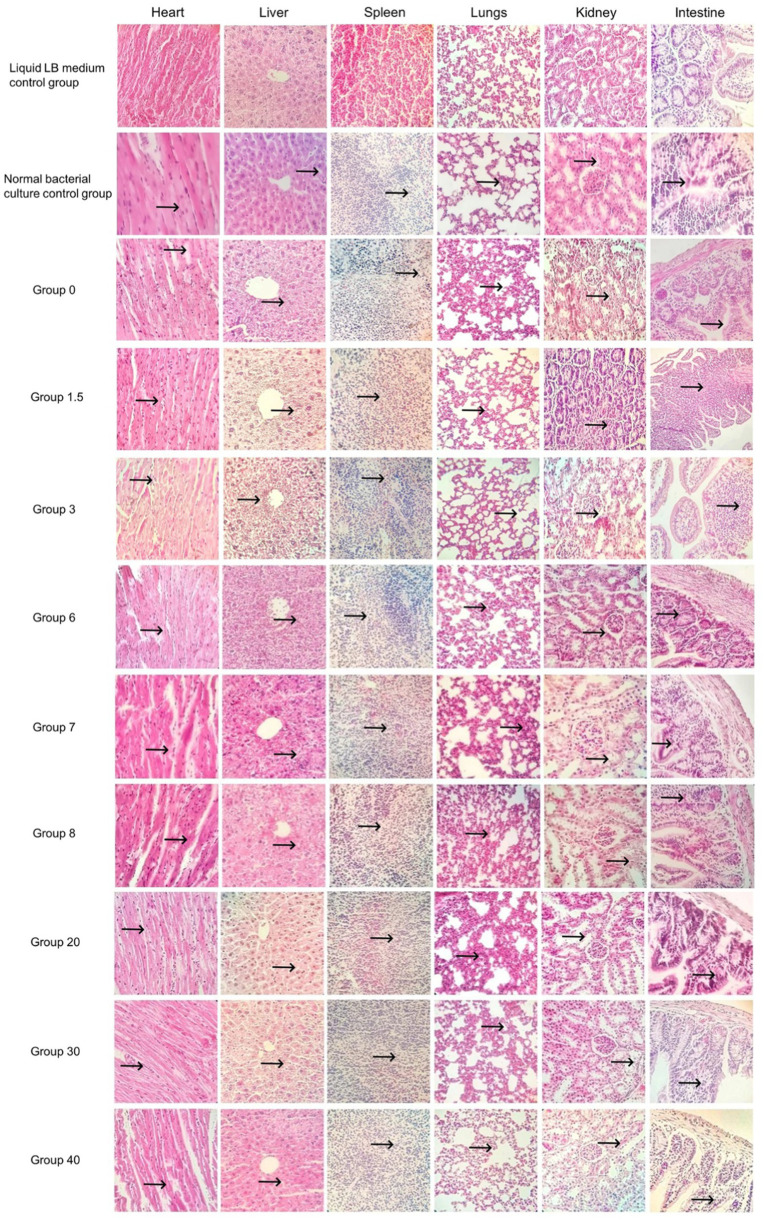
Pathological sections of the control group and the single-factor environmental stress group (400×).

**Figure 5:**
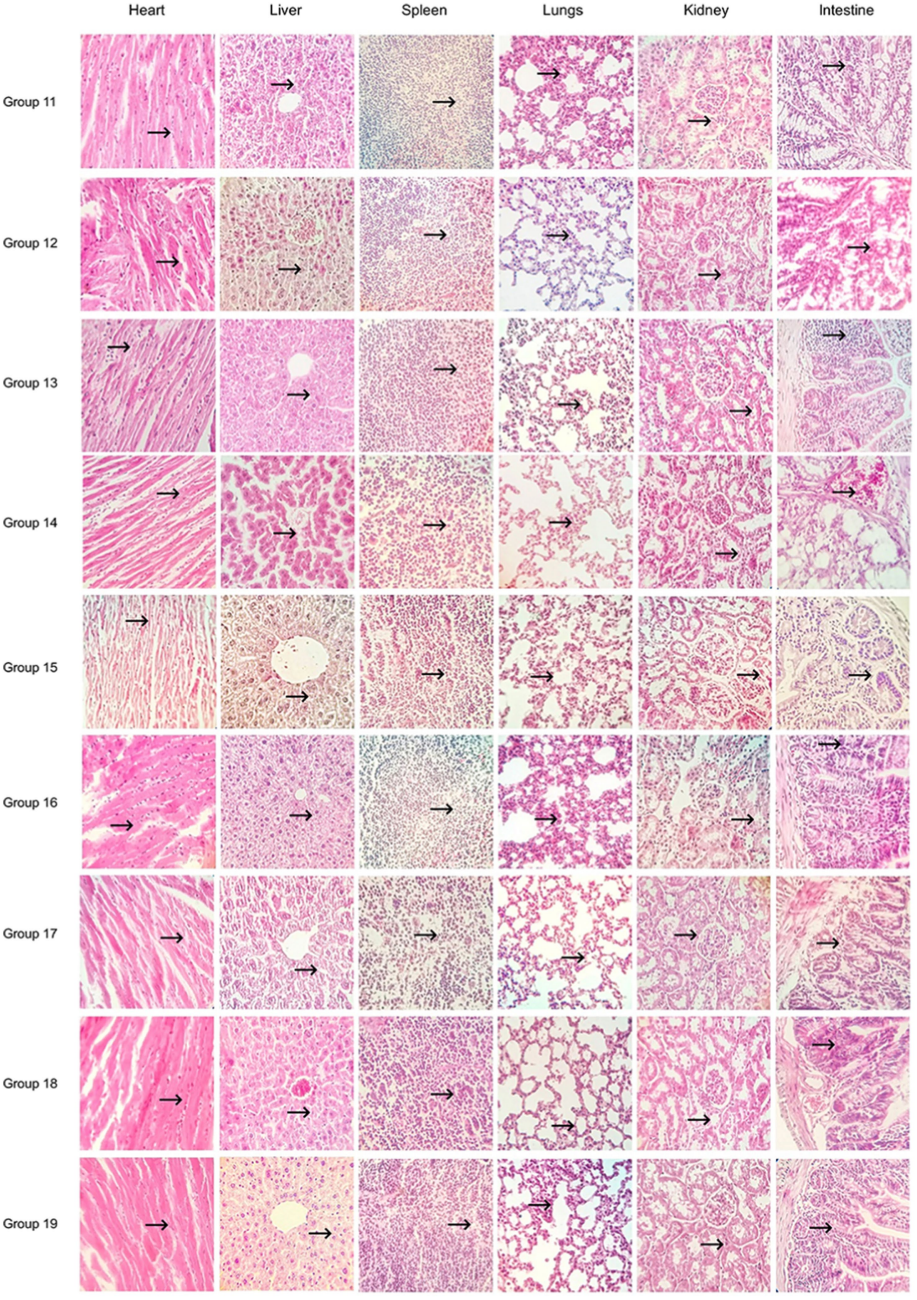
Multifactorial environmental stress factor group pathology sections (400×).

The results of the pathological sections (as shown in Figures 4, 5) showed a small differences in the pathological changes in the organs of the mice in each group. We repeated the histopathological profiles shown in 10 mice included in each group of studies. Mice in the bacterial culture injection group showed more severe pathological damage than mice in the normal control group. It can be seen that the myocardial fibers of the mice in the bacterial injection group were swollen and broken, with inflammatory cell infiltration between the myocardial fibers and disappearance of myocardial cell nuclei. Widening of the hepatic blood sinusoids, narrowing of the central vein, necrosis of individual hepatocytes, loss of nucleolysis. Necrosis and disappearance of lymphocytes in some areas of the red medulla of the spleen. A few alveolar lumens appear dilated, with dilated capillaries in the alveolar walls and massive leukocytosis. Proliferation of glomerular mesangial cells, narrowing of the lumen of dense plaques, detachment and necrosis of tubular epithelial cells. The mucosal epithelium of the small intestine was detached and individual intestinal glandular cells were necrotic and disappeared.

## 4 Discussion

*B. cereus* produces a variety of virulence factors to participate in the pathogenic process of bacteria (Mazzantini et al., 2020). *B. cereus* also forms endospores and biofilms to help *B. cereus* adapt to harsh environments (Bressuire-Isoard et al., 2018; Caro-Astorga et al., 2020). The bovine lethal *B. cereus* used in this experiment harbors multiple virulence genes (e.g., *hblA*, *hblC*, and *hblD*). In this study, a preliminary study of four virulence genes (*nheA*, *hblD*, *cytK*, and *entFM*) of *B. cereus* was conducted. More in-depth studies are needed in the future to determine the interaction patterns of proteins associated with *B. cereus* virulence genes, with a view to determining, at the molecular level, the reasons why environmental stressors alter the expression of *B. cereus* virulence genes. More virulence genes need to be studied to further understand the pathogenic mechanism of *B. cereus* for better prevention and control of *B. cereus* disease.

The influence of different factors on bacterial growth and pathogenicity is very complex and therefore appropriate strategies need to be developed to prevent and control bacterial infections (Hosseini et al., 2024; Ehling-Schulz et al., 2015). Changes in temperature, pH and salt concentration all affect the survival of *B. cereus* and the expression of its virulence genes. When temperature was used as a univariate variable, the results showed that high temperature inhibited the expression of *B. cereus* virulence genes. However, the control of *B. cereus* bacterial disease has limitations if it is done by temperature alone (High-temperature sterilization methods cannot be used in some cases, e.g., temperature-sensitive plastics). The use of 20°C and 30°C conditions was not effective in controlling *B. cereus* disease, while the use of 40°C conditions to control *B. cereus* disease may result in the development of heat resistance in the bacteria. In summary, *B. cereus* disease cannot be controlled by temperature alone, but temperature can be used as an aid to accomplish some of the disinfection. When pH was used as a variable it was evident that the bacterium had very low expression at pH 8.0, with the most prominent changes in *nheA*, *cytK* and *entFM*. High pH can be used to control *B. cereus* when pH is used as a one-way variable. When salt concentration was used as a variable (at salt concentrations of 0, 1.5, and 3%), all virulence genes showed degrees of low expression compared to the control (the salt concentration in the control group was 1%). Control of *B. cereus* can be achieved to a certain extent by a single stressor, but prolonged use of a single stressor may lead to the development of tolerance in *B. cereus*. Under multiple environmental stressors, *nheA*, *hblD* and *cytK* were least expressed at a temperature of 40°C, pH 6.0, and a salt concentration of 3.0%, and *entFM* was least expressed at a temperature of 20°C, pH 8.0, and a salt concentration of 1.5%. It is evident that different stress conditions inhibit the expression of *B. cereus* virulence genes (*nheA*, *cytK*, *hblD*, *entFM*). The time of death of mice indicated that bacteria cultured at pH 6.0 and 7.0 were relatively more virulent, and those cultured at 20 and 30°C were more virulent; among the multifactorial groups, *B. cereus* cultured in groups 11 (high temperature and high pH), 15 (medium temperature and high pH and high salt), 17 (medium temperature and neutral pH), and 18 (medium temperature and low pH and medium salt concentration) were relatively more virulent. In summary, it can be seen that different environmental stressors coercion may result in the expression of virulence genes and pathogenicity of *B. cereus* being affected. A single virulence gene showing low expression may not attenuate the pathogenicity of *B. cereus*, or even low expression of some virulence genes may enhance the pathogenicity of *B. cereus*. This may be due to the relatively complex pathogenic mechanisms of *B. cereus* (Multiple genes collectively affect the virulence of *B. cereus*; weak expression of a single gene may not affect the virulence of *B. cereus*). The specific reasons for these phenomena need to be further studied. In the future, there is a need to find suitable stress conditions to suppress the expression of virulence genes and the pathogenicity of strains of *B. cereus*.

The results of Wang et al. were significantly different from those of this paper (different expression of virulence genes) (Wang and Liu, 2018). It is possible that the isolation environments of the two strains were different or the biological characteristics of the strains were somewhat different, which led to different expression of virulence genes in the two bacterial strains. A study by and others showed that pu-erh tea broth significantly reduced the expression levels of *cytK*, *nheA* and *hblD* genes and attenuated the damage caused by *B. cereus* to the liver cells of mice (Sha, 2018). This result is consistent with the findings of the present study, suggesting that inhibition of virulence gene expression is one of the effective ways to reduce the pathogenicity of *B. cereus*. To prevent the development of resistance in *B. cereus*, the use of antibiotics is severely restricted (Zheng et al., 2024). Therefore, it is clinically relevant to explore the control of environmental stressors to accomplish food-handling-related appliances and farm environmental decontamination. The method used in this study reduces the probability of human and animal infection with *B. cereus* disease by decreasing *B. cereus* virulence gene expression. The method used in this study is non-toxic and is expected to be used in the future for the disinfection of utensils related to food handling (kitchen utensils, slaughterhouse equipment) and the environment of farms. Both pharmaceutical and chemical disinfection methods can produce residues and chemical disinfection has a high odor; this makes it unsuitable for sterilizing some utensils and environments (kitchenware, slaughterhouse equipment and farm environments) (Wildenthal et al., 2023; Doyle et al., 2021). Bacteriophages have been effective in the control of *B. cereus* disease (Huang et al., 2024). However, bacteriophages may affect the growth of beneficial bacteria when used, thereby disrupting the flora balance of the environment. It is also possible that bacteriophages could lead to some resistance to *B. cereus*, which would increase the cost of *B. cereus* disease prevention and control (bacteriophage enhancement or replacement with new phages is needed to achieve control of *B. cereus* disease). The potential safety risks of bacteriophages in use need to be further evaluated. The method used in this study does not produce residues and is relatively safe to operate. It is believed that with further research, environmental stressors can be gradually used in the future to replace antibiotics to reduce *B. cereus* in the environment. New acidic disinfectants can be developed to accomplish disinfection, and the salt concentration in the disinfectant can be increased appropriately to enhance its effectiveness. This study provides a reference for the development of new disinfectants and the prevention and control of bacterial diseases of *B. cereus*.

## 5 Conclusion

Environmental stressors (temperature, salt concentration, acidity and alkalinity) have an effect on the expression of four virulence genes (*nheA*, *cytK*, *hblD*, *entFM*) in *B. cereus*. Under a single environmental stressors, *nheA*, *hblD*, *cytK* and *entFM* had the lowest expression at 40°C, pH 8.0, and were lowly expressed at all salt concentrations except the control group. Under multiple environmental stressors, *nheA*, *hblD* and *cytK* were least expressed at a temperature of 40°C, pH 6.0, and salt concentration of 3.0%, and *entFM* was least expressed at a temperature of 20°C, pH 8.0, and salt concentration of 1.5%. New disinfectants can be developed to control the spread of the bacteria based on the above results (focusing on the development of acidic disinfectants, while the salt concentration in the disinfectant can be increased appropriately).

## Data Availability

The datasets presented in this study can be found in online repositories. The names of the repository/repositories and accession number(s) can be found at: https://www.ncbi.nlm.nih.gov/genbank/, CP129005; https://www.ncbi.nlm.nih.gov/genbank/, CP129006.
